# Clinical predictors of pathological femoral fractures in children: a retrospective comparative study

**DOI:** 10.1186/s10195-026-00954-2

**Published:** 2026-07-18

**Authors:** Tanja Kraus, Clemens Clar, Jürgen Rünk, Patrick Reinbacher, Vanessa Sackl, Georg Singer, Magdalena Rusch, Andreas Leithner

**Affiliations:** 1 Department of Orthopaedics and Trauma, Pediatric Orthopedic Unit, Medical University of Graz, Auenbruggerplatz 5, 8036 Graz, Austria; 2 Department of Paediatric Surgery, Tartu University Hospital, Tartu, Estonia; 3 Department of Paediatric and Adolescent Surgery, Medical University of Graz, Graz, Austria

**Keywords:** pathological fracture, pediatric femur fracture, pediatric fracture

## Abstract

**Background:**

Femoral fractures in children are uncommon and are usually trauma-related; however, some occur secondary to underlying bone pathology. Early differentiation between traumatic and pathological fractures is essential to avoid delayed diagnosis. This study aimed to identify clinical predictors associated with pathological femoral fractures in children.

**Material and methods:**

In this retrospective single-center comparative study, 432 pediatric patients (0–17 years) with femoral fractures were included. These fractures were classified as traumatic (*n* = 386, 89.4%; treated between 2011 and 2023) or pathological (*n* = 46, 10.6%; treated between 2002 and 2021) on the basis of radiological findings, advanced imaging, or histopathological confirmation. Demographic, anatomical, and injury-related variables were compared between the two groups using Mann–Whitney *U* or chi square tests.

**Results:**

Patients with pathological fractures tended to be older (10.1 ± 4.3 years versus 8.3 ± 5.9 years, *p* = 0.090) and age-group distribution differed significantly between the two groups (*p* < 0.001). Traumatic fractures showed a bimodal age distribution, whereas pathological fractures followed a unimodal pattern. Both groups most commonly sustained diaphyseal fractures (58.7% pathological versus 59.8% traumatic). Low-energy trauma mechanisms were significantly more common in pathological fractures (78.9% versus 49.5%, *p* = 0.001).

**Conclusions:**

Pathological femoral fractures in children differ from traumatic fractures with respect to age distribution, anatomical location, and injury mechanism. They are more likely to occur following low-energy trauma and demonstrate a different age distribution to traumatic fractures. Careful assessment of patient age, fracture location, and trauma mechanism may aid in the early identification of underlying bone pathology and support appropriate diagnostic evaluation.

## Introduction

Bone fractures are among the leading causes of morbidity during childhood and adolescence worldwide, despite advances in prevention, prehospital care, and surgical treatment [1–4]. Although pediatric bone has substantial remodeling potential, fractures may still result in pain, hospitalization, reduced mobility, deformity, and limitations in daily activities during critical periods of growth and development [5–7].

Femoral fractures account for approximately 1.4–1.7% of all pediatric fractures and are, therefore, relatively uncommon. However, they represent the leading cause of prolonged hospitalization among injured children [8, 9]. These fractures occur more frequently in males and demonstrate a well-established bimodal age distribution, with incidence peaks in early childhood and late adolescence. The femoral diaphysis is the most commonly affected anatomical region. Injury mechanisms vary by age, with low-energy falls predominating in younger children and high-energy trauma, including traffic-related accidents and sports injuries, occurring more frequently in adolescents [8–11].

Pathological femoral fractures occur in bone weakened by underlying local or systemic disease, including benign or malignant tumors, metabolic bone disorders, or other structural abnormalities. Differentiating pathological from traumatic fractures is clinically important because diagnostic evaluation, treatment strategies, and prognosis differ substantially. In contrast to traumatic fractures, pathological fractures may occur after minimal trauma and may present with atypical fracture patterns or anatomical locations.

Despite their clinical importance, early identification of pathological femoral fractures remains challenging in routine practice. Clinical history may provide suggestive indicators, such as low-energy trauma or preceding pain, whereas radiographic findings may initially be subtle or inconclusive. Consequently, advanced imaging modalities, including computed tomography (CT) and magnetic resonance imaging (MRI), are often required for definitive diagnosis. At present, no standardized clinical criteria reliably distinguish pathological from traumatic femoral fractures in pediatric patients, and diagnostic decision-making frequently depends on individual clinical judgment rather than evidence-based predictors [12–14].

Therefore, the aim of this study was to identify clinical and injury-related predictors associated with pathological femoral fractures in children by systematically comparing them with traumatic femoral fractures. Specifically, we evaluated whether patient age, fracture location, and injury mechanism differ between the two groups and may support early recognition of underlying bone pathology.

## Patients and methods

### Study design and ethical approval

This retrospective single-center observational study analyzed pediatric patients with femoral fractures treated at a level I trauma center and pediatric referral hospital in Central Europe. The overall study period ranged from April 2002 to October 2023. Traumatic femoral fractures were identified between September 2011 and October 2023, whereas pathological femoral fractures were identified between April 2002 and December 2021. The observation period for pathological fractures was extended because of their substantially lower incidence in the pediatric population, allowing inclusion of a sufficiently large cohort for comparative analysis. By contrast, the traumatic fracture cohort was restricted to the period with complete electronic documentation available at our institution.

The study was conducted in accordance with the principles of the Declaration of Helsinki and approved by the Ethics Committee of the Medical University of Graz (EK: 34-350 ex 21/22 and 35-475 ex 22/23). Owing to the retrospective study design and pseudonymized data analysis, the requirement for informed consent was waived according to institutional regulations. All data were pseudonymized before analysis in accordance with institutional and legal data protection requirements.

#### Patient selection and eligibility criteria

All pediatric patients aged 0–17 years with a radiologically confirmed femoral fracture treated at the participating institution during the predefined study periods were screened for eligibility. Fractures were identified at the time of hospital admission using plain radiographs and/or computed tomography (CT). Magnetic resonance imaging (MRI) was additionally performed when clinically indicated. Imaging studies were reviewed by a board-certified radiologist and/or the treating pediatric surgeon or orthopedic surgeon. Patients were excluded if the fracture did not involve the femur or if radiological or clinical documentation was incomplete.

Femoral fractures were classified as either traumatic or pathological. Traumatic fractures were defined as fractures occurring in previously healthy bone following an adequate traumatic event. Pathological fractures were defined as fractures occurring in bone weakened by an underlying local or systemic pathology, including benign or malignant bone tumors, metabolic bone disease, or other conditions compromising bone integrity. Classification as pathological was based on radiological findings and, where clinically indicated, confirmed by advanced imaging and/or histopathological analysis.

After application of the inclusion and exclusion criteria, a total of 432 pediatric patients were included in the final analysis, comprising 386 traumatic and 46 pathological femoral fractures.

#### Data retrieval and study variables

Clinical and radiological data were retrieved from the institutional electronic health information system (MEDocs). Data extraction was independently performed by four investigators (P.R., V.S., M.R., and C.C.) using standardized data collection procedures to minimize extraction bias and improve data consistency. Any discrepancies identified during data collection were resolved by joint review and consensus.

Collected variables included demographic characteristics (sex, date of birth, and age at injury), injury mechanisms, fracture classification, anatomical localization, fracture side, and radiological findings at the time of presentation. Imaging studies, including plain radiographs, CT, and MRI where applicable, were reviewed in conjunction with the corresponding clinical records.

Patients were stratified into predefined age groups to account for developmental differences: infants (< 1 year), preschool children (> 6 years), prepubertal school-aged children (> 10 years), early adolescents (> 14 years), and late adolescents (> 17 years).

#### Measurements and outcomes

Injury mechanisms were dichotomized into low- and high-energy trauma. Low-energy trauma included falls on level ground, falls from heights < 3 m and comparable low-impact mechanisms. High-energy trauma included falls from heights > 3 m, crush or direct-impact injuries, and traffic-related accidents. Miscellaneous injury mechanisms comprised cases in which the mechanism of injury was unclear, atypical, or insufficiently documented.

All fractures were classified according to their anatomical localization as proximal, diaphyseal, or distal femoral fractures. In addition, fractures involving the growth plate were further subclassified according to the Salter–Harris classification system. The primary study objective was to compare fracture localization between traumatic and pathological femoral fractures across predefined age groups. Secondary outcome measures included demographic characteristics, injury mechanisms, fracture patterns, and radiological characteristics associated with pathological femoral fractures.

#### Statistical analysis

The dataset was compiled using Excel*®* (Microsoft*®* Corporation, Redmond, WA, USA) and statistical analyses were subsequently performed with IBM SPSS Statistics, version 29 (IBM Corp., Armonk, NY, USA).

Continuous variables are summarized as means ± standard deviations. Categorical variables are reported as absolute numbers and percentages. Selected estimates are presented with 95% confidence intervals.

Comparisons between traumatic and pathological fracture cohorts were conducted using Pearson chi-square tests for categorical variables and Mann–Whitney *U* tests for continuous variables. Effect sizes for categorical group comparisons were quantified using Cramér’s *V*. All statistical tests were two-sided, and a *p* value < 0.05 was considered statistically significant.

Because of the retrospective exploratory study design and the low incidence of pathological femoral fractures in the pediatric population, a formal a priori sample size calculation was not feasible. Therefore, all eligible patients treated during the predefined study periods were included in the final analysis. Owing to the limited number of pathological fractures, multivariable analyses were not performed. Missing data were handled using complete-case analysis. Analyses were performed using all available observations for each variable, and missing values were not imputed.

## Results

A total of 432 pediatric femoral fractures were included in the analysis, comprising 386 traumatic fractures (89.4%; 95% CI 86.1–91.9%) and 46 pathological fractures (10.6%; 95% CI 8.1–13.9%). Sex distribution did not differ significantly between cohorts, with male patients accounting for 68.1% in the traumatic group and 65.2% in the pathological group (*p* = 0.815, chi-square test). Fracture laterality also did not differ significantly between groups (*p* = 0.889, chi-square test, Cramér’s *V* = 0.023) (Table 1).

**Table 1:** Overview of key differences in demographic, anatomical, and mechanistic patterns between traumatic and pathological femoral fractures

Parameter	Traumatic fractures (*n* = 386)	Pathological fractures (*n* = 46)	Statistics
Sex	Male: 263 (68.1%) Female: 123 (31.9%)	Male: 30 (65.2%) Female: 16 (34.8%)	*p* = 0.815 (chi-square test)
Side of injury	Left: 214 (55.5%) Right: 170 (44.0%) Bilateral: 2 (0.5%)	Left: 25 (54.3%) Right: 21 (45.7%) Bilateral: 0 (0%)	***p* = 0.889,** (chi-square test) **Cramér’s *V* = 0.023**
Most common anatomical location	Proximal: 41 (10.6%) Diaphysis: 231 (59.8%) Distal: 114 (29.6%)	Proximal: 11 (23.9%) Diaphysis: 27 (58.7%) Distal: 8 (17.4%)	*p* = 0.017 (chi-square test) Cramér’s *V* = 0.138
Growth plate relation (Salter–Harris)	Total = 34 Proximal: 0 Distal: 34	Total = 4 Proximal: 2 Distal:2	
Age distribution (peak)	Bimodal: Early childhood (age 2 years) & Late adolescence (age 15 years)	Unimodal: Late adolescence (age 15 years)	
Mean age (years)	8.3 ± 5.9 years; 95% CI 7.7–8.9	10.1 ± 4.3 years; 95% CI 8.8–11.4	***p* = 0.090, Mann–Whitney *U* test**
Mean age by location	Proximal: 10.83 ± 4.90 years Diaphyseal: 7.94 ± 5.74 years Distal: 8.24 ± 6.20 years	Proximal: 7.20 ± 4.99 years Diaphyseal: 11.26 ± 3.65 years Distal: 11.15 ± 2.88 years	Proximal: *p* = 0.048 Diaphyseal: *p* < 0.001 Distal: *p* = 0.029 Mann–Whitney-*U* test
Most common setting	Home: 135 (35.0%) Sports/play activities: 135 (35.0%) Traffic-related: 100 (25.9%) Educational institutions: 14 (3.6%) No clearly recalled accident: 2 (0.5%)	Sports/play activities: 19 (41.3%) No remembered trauma: 11 (23.9%) Home: 8 (17.4%) Educational institutions: 5 (10.9%) Traffic-related settings: 3 (6.5%)	*p* < 0.001 (chi-square test) **Cramér’s *V* = 0.423**
Injury mechanisms	Traffic: 100 (25.9%) Fall height (< 3 m): 89 (23.1%) Crush injury: 85 (22.0%) Fall on level ground: 65 (16.8%) Miscellaneous: 37 (9.6%) Fall height > 3 m: 10 (2.6%)	Falls on level ground: 16 (34.8%) Falls < 3 m: 5 (10.9%) Crush injuries: 4 (8.7%) High falls > 3 m: 3 (6.5%) Traffic: 1 (2.2%) Miscellaneous: 17 (37.0%)	*p* < 0.001 (chi-square test) **Cramér’s *V* = 0.17**
Energy level of injury mechanism	Low-energy: 191 (49.5%) High-energy: 195 (50.5%)	Low-energy: 30 (78.9%) High-energy: 8 (21.1%) Missing: 8	*p* = 0.001 (chi-square test) Cramér’s *V* = 0.277
Most common etiology	Trauma (386)	Bone cysts (12), osteoporosis* (10), osteosarcoma (6), nonossifying fibroma (NOF) (5), fibrous dysplasia (3), osteomyelitis (3), Ewing sarcoma (1), osteogenesis imperfecta (1), eosinophilic granuloma (1), chondrosarcoma (1), NOF + bone cyst (1), stress fracture (1), neuroblastoma (1)	

^*^osteoporosis due to immobility (e.g. in patients with cerebral palsy)

Of the 386 traumatic femoral fractures, 41 occurred in the proximal femur (10.6%), 231 were located in the diaphyseal region (59.8%), and 114 affected the distal femur (29.6%). None of the proximal femoral fractures involved the proximal femoral physis. By contrast, 34 of the 114 distal fractures (29.8%) involved the distal femoral physis. Within this subgroup, Salter–Harris type II fractures were the most common (*n* = 22; 64.7%), followed by type III fractures (*n* = 8; 23.5%), type I fractures (*n* = 3; 8.8%), and type IV fractures (*n* = 1; 2.9%).

Of the 46 pathological femoral fractures, 11 involved the proximal femur (23.9%), 27 occurred in the diaphyseal region (58.7%), and 8 affected the distal femur (17.4%). Of the 11 proximal fractures, 2 (18.2%) were classified as Salter–Harris type II injuries, whereas two of the eight distal fractures could be classified as Salter–Harris type I (*n* = 1, 12.5%) and type II (*n* = 1, 12.5%) fractures.

**Fig. 1:**
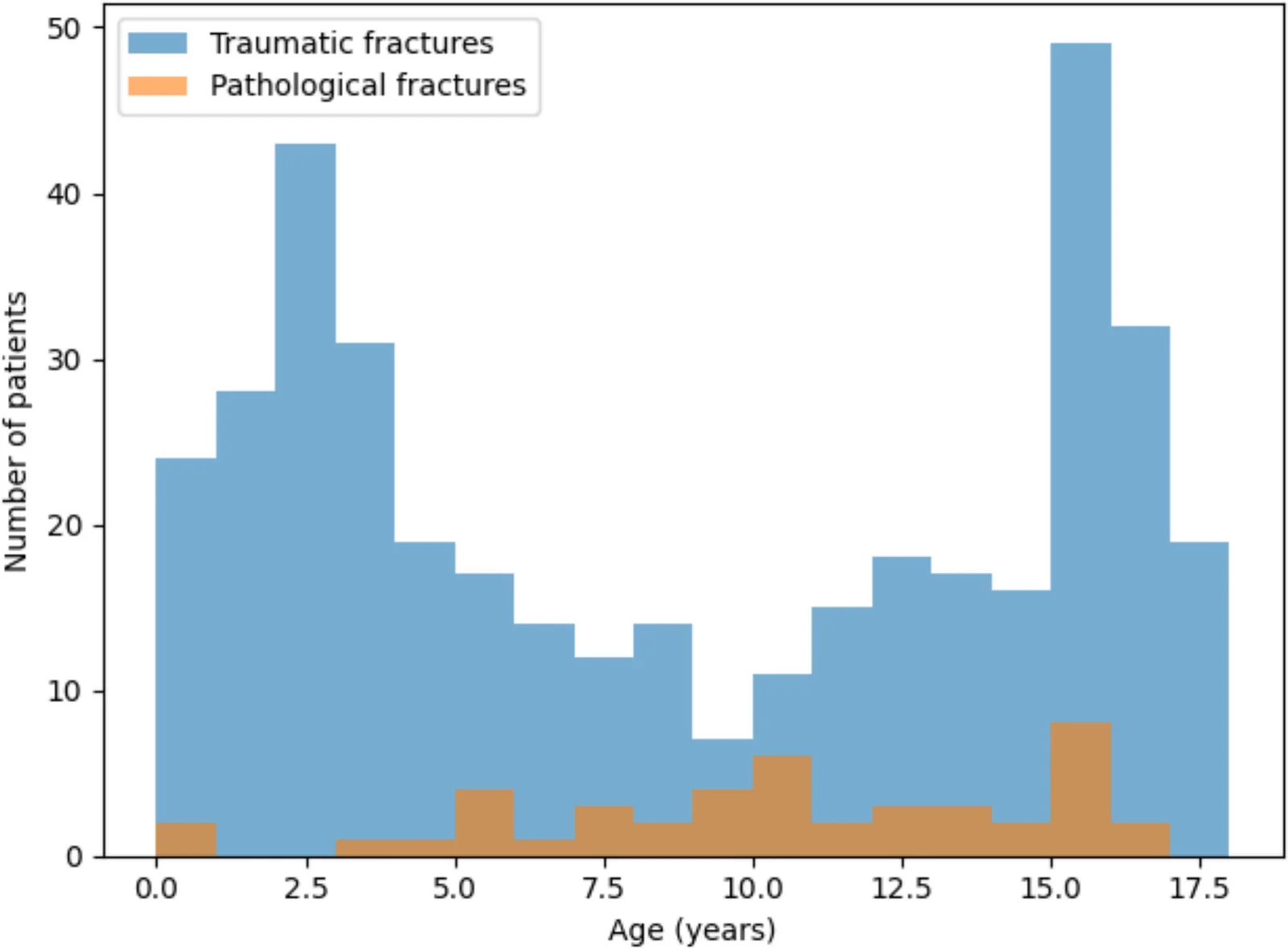
Age distribution of traumatic and pathological fractures. Traumatic fractures demonstrated a bimodal age distribution with peaks in early childhood and adolescence, whereas pathological fractures showed a predominantly unimodal distribution with a peak in mid-childhood

Patients with pathological fractures were older than those with traumatic fractures, although this difference did not reach statistical significance (mean age 10.1 ± 4.3 years; 95% CI 8.8–11.4 versus 8.3 ± 5.9 years; 95% CI 7.7–8.9; *p* = 0.090, Mann–Whitney *U* test). Traumatic femoral fractures demonstrated a clear bimodal age distribution, with incidence peaks at approximately 2 and 15 years of age. By contrast, pathological fractures showed a predominantly unimodal age distribution with a peak at 15 years (Fig. 1).

**Fig. 2:**
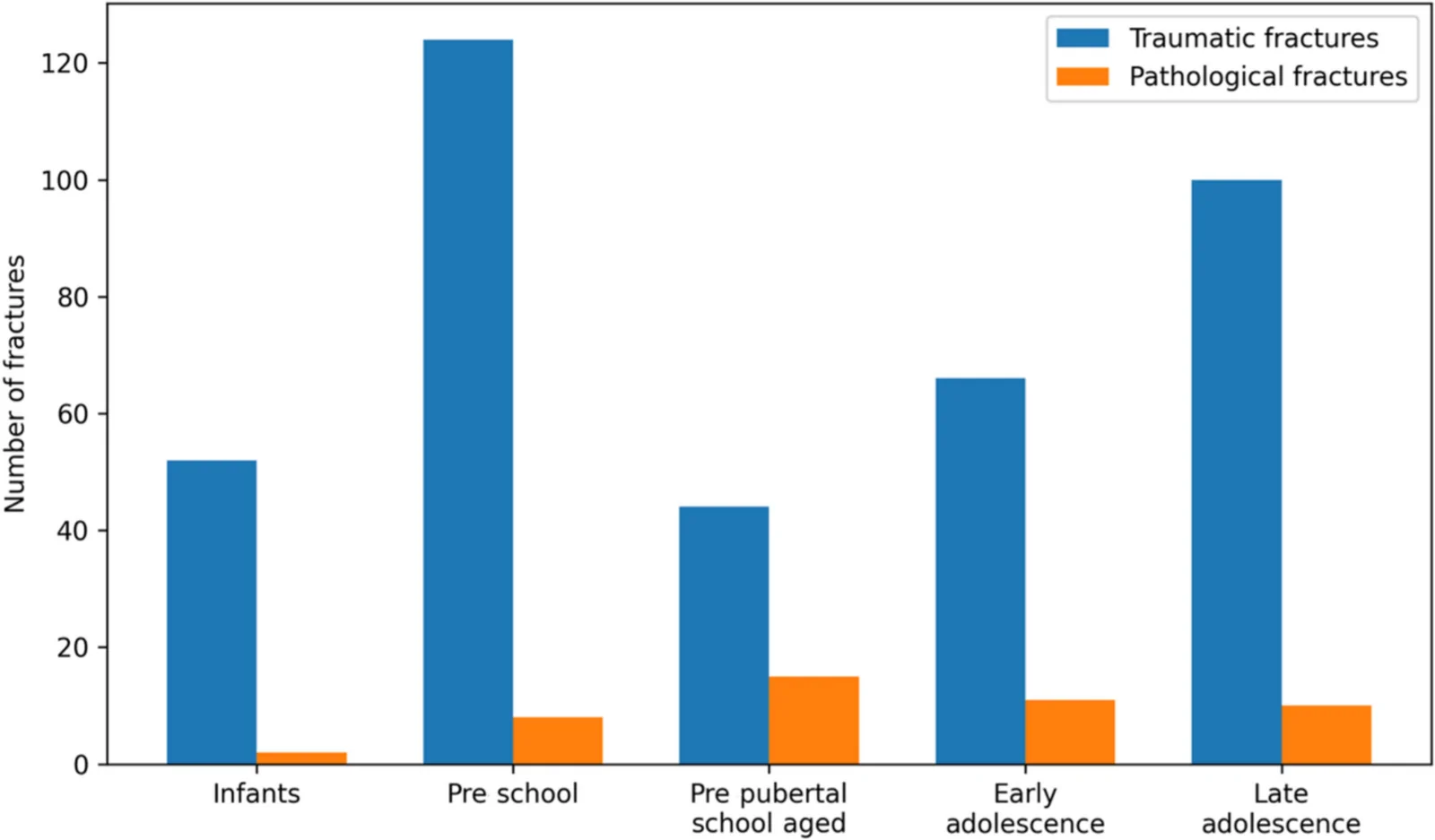
Distribution of traumatic and pathological pediatric femoral fractures across predefined age groups

When stratified into the predefined age groups, the distribution differed significantly between traumatic and pathological fractures (*p* < 0.001, chi-square test), with a moderate effect size (Cramér’s *V* = 0.22). Traumatic fractures occurred most frequently in preschool children, whereas pathological fractures were most frequently observed in prepubertal school-aged children (Fig. 2).

Regarding injury settings, traumatic fractures most frequently occurred at home and during sports or play activities, whereas pathological fractures occurred most commonly during sports or play activities. The distribution of injury settings differed significantly (*p* = 0.001, chi-square test) and showed a moderate-to-large effect size (Cramér’s *V* = 0.423) (Table 1).

Among traumatic fractures, traffic accidents represented the most frequently documented specific injury mechanism (Table 1). Among pathological fractures with available mechanism data, the majority resulted from falls on level ground or unspecified mechanisms. The distribution of specific injury mechanisms differed significantly between traumatic and pathological fractures (*p* < 0.001, chi-square test, Cramér’s *V* = 0.277).

When injury mechanisms were categorized into low- and high-energy injury mechanisms, pathological fractures were significantly more likely to result from low-energy mechanisms compared with traumatic fractures (78.9%; 95% CI 63.7–88.9% versus 49.5%; 95% CI 44.5–54.5%; *p* = 0.001, chi-square test). This association demonstrated a small-to-moderate effect size (Cramér’s *V* = 0.17).

The most common underlying etiologies of pathological fractures were bone cysts (*n* = 12, 26.1%), osteoporosis (*n* = 10, 21.7%), and osteosarcoma (*n* = 6, 13.0%). Less frequent causes included nonossifying fibroma (*n* = 5, 10.9%), fibrous dysplasia (*n* = 3, 6.5%), and osteomyelitis (*n* = 3, 6.5%). For more details see Table 1.

Mean ages across anatomical localizations significantly differed in both cohorts (Table 1). In the traumatic fracture group, mean age was highest for proximal femoral fractures (10.83 ± 4.90 years), followed by distal (8.24 ± 6.20 years) and diaphyseal fractures (7.94 ± 5.74 years). In the pathological fracture group, mean age was 7.20 ± 4.99 years in patients with proximal fractures, 11.26 ± 3.65 years for diaphyseal fractures, and 11.15 ± 2.88 years in the distal femoral fracture group.

## Discussion

Despite the clinical importance of pathological femoral fractures in children, evidence regarding potential clinical predictors for their early identification remains limited. Most currently available studies focus primarily on treatment strategies or specific underlying pathologies such as benign bone cysts, osteosarcoma or metabolic bone disease rather than on comparative clinical characteristics distinguishing pathological from traumatic fractures [12, 16, 17, 21]. Consequently, standardized diagnostic criteria for the early recognition of pathological fractures in pediatric patients are lacking, and diagnostic decision-making in routine clinical practice frequently relies on individual clinical experience and radiographic interpretation. This represents a relevant area of uncertainty because early radiographic findings may initially be subtle or nonspecific, particularly in children presenting after apparently minor trauma [12, 17, 21]. Furthermore, the rarity and heterogeneity of pathological fractures in the pediatric population limit the availability of large comparative studies and complicate the establishment of evidence-based diagnostic algorithms [16, 17]. Therefore, identification of reproducible clinical indicators that may increase suspicion for underlying bone pathology remains clinically important.

The aim of this study was to identify clinical and history-based features that distinguish pathological femoral fractures from traumatic femoral fractures in a pediatric population. Our findings demonstrate that pathological fractures differ from traumatic fractures with respect to patient age distribution, injury mechanism, and anatomical location. In particular, pathological femoral fractures were more frequently observed in prepubertal and adolescent patients, were most frequently located in the diaphyseal femoral segment, and were significantly more often associated with low-energy or incongruent trauma mechanisms.

A pronounced bimodal age distribution was observed in the traumatic fracture cohort, with incidence peaks in early childhood and late adolescence. This pattern is well established in the literature and reflects age-related exposure to different injury mechanisms, motor development, and risk-taking behavior [2, 15]. The results are consistent with previous population-based studies by Hinton et al.[15] and Engström et al.[9], which reported similar incidence peaks and a higher prevalence of femoral fractures among males. By contrast, pathological fractures in this cohort were more frequently observed in older children and adolescents. This may reflect the increased vulnerability of structurally compromised bone during periods of rapid growth, when mechanical stress on the femur is high and underlying lesions may become clinically apparent [15–17].

With regard to anatomical distribution, pathological fractures in our cohort were most commonly located in the diaphyseal femoral segment. This pattern is in line with previous reports indicating that several frequent pediatric benign tumor-like lesions, including cystic entities, predominantly involve the medullary cavity of long bones and may, therefore, present with diaphyseal fractures in areas of substantial biomechanical loading [15–18]. Importantly, however, the anatomical predilection differs substantially depending on the underlying diagnosis: while various benign lesions may occur in diaphyseal regions, primary malignant bone tumors such as osteosarcoma classically arise in the metaphysis adjacent to the growth plate, most commonly around the knee. In this context, the near-exclusive occurrence of epiphyseal and combined growth plate-related fractures within the pathological cohort, despite their overall rarity, is clinically relevant and underscores that atypical fracture locations, particularly epiphyseal, metaphyseal, or physeal involvement, should prompt a high index of suspicion for underlying bone pathology, especially when fractures occur after low-energy or incongruent trauma mechanisms [19, 20].

In skeletally immature patients, fractures involving open growth plates are typically expected to follow Salter–Harris patterns after acute trauma. By contrast, most pathological fractures in this cohort could not be classified according to the Salter–Harris system despite open physes. This finding is consistent with the concept that pathological fractures arise from intrinsic bone weakness rather than acute physeal injury mechanisms, leading to fracture propagation through adjacent metaphyseal or diaphyseal bone [12]. The absence of typical Salter–Harris fracture patterns in the presence of open growth plates may, therefore, represent an additional radiographic indicator of underlying bone pathology, as observed in the present study.

A further key differentiating feature was the discrepancy between the reported injury mechanism and fracture severity. Traumatic femoral fractures were predominantly associated with high-energy trauma, whereas pathological fractures were most often caused by low-energy events or occurred without a clearly recalled traumatic trigger. This incongruity between injury mechanism and fracture pattern has been consistently described as an important clinical warning sign and reinforces the diagnostic value of a thorough and critical assessment of the injury history [17, 18, 21].

An additional methodological aspect that should be considered when interpreting our findings is the difference in observation periods between traumatic and pathological femoral fractures. This distinction was intentional and reflects the substantially lower incidence of pathological fractures in the pediatric population. Extending the observation period for pathological fractures was necessary to obtain a sufficiently large and clinically meaningful cohort for comparative analysis. Importantly, the objective of this study was not to compare temporal incidence trends but rather to identify differences in clinical and injury-related characteristics between pathological and traumatic fractures. Therefore, the differing observation periods are unlikely to have materially influenced the comparative assessment of fracture characteristics, as the analyzed variables—such as patient age, anatomical location, and injury mechanism—represent intrinsic clinical features independent of the specific time frame in which the fractures occurred. Nevertheless, this methodological difference should be acknowledged when interpreting the results.

Taken together, these findings suggest that a combination of patient age, fracture location and pattern, and injury mechanism provides meaningful contextual information that may assist clinicians in differentiating pathological from traumatic femoral fractures, and in identifying patients who warrant further diagnostic evaluation. Early recognition of pathological fractures is essential to avoid delayed diagnosis of underlying disease, initiate appropriate treatment, and reduce the risk of further skeletal complications.

This study has several limitations. The retrospective design introduces the potential for selection bias, incomplete documentation, and misclassification, particularly in cases where advanced imaging was not uniformly available. Furthermore, the single-center setting may limit generalizability to other populations and healthcare systems. Although systematic data extraction and independent review were performed to minimize bias, reliance on medical records may have resulted in incomplete or imprecise documentation of injury mechanisms in some cases. In addition, the relatively small number of pathological fractures limited statistical power and precluded multivariate analysis, restricting the ability to adjust for potential confounders and identify independent predictors. Accordingly, the results should be interpreted as exploratory and hypothesis-generating rather than definitive evidence of causality.

In conclusion, pathological femoral fractures in children differ from traumatic fractures with respect to age distribution, anatomical location, and injury mechanism. They occur more frequently in older pediatric patients, are commonly located in the femoral diaphysis, and are significantly associated with low-energy or incongruent trauma mechanisms. A mismatch between fracture severity and injury mechanism, as well as atypical fracture locations or non-Salter–Harris fracture patterns despite open physes, should raise suspicion for underlying bone pathology. Consideration of these clinical and radiographic features may support earlier diagnosis and appropriate diagnostic evaluation in pediatric patients with femoral fractures.

## Data Availability

The data that support the findings of this study are not openly available owing to reasons of sensitivity but are available from the corresponding author upon reasonable request.
